# Risk Factors for Buruli Ulcer, Benin

**DOI:** 10.3201/eid1209.050598

**Published:** 2006-09

**Authors:** Martine Debacker, Françoise Portaels, Julia Aguiar, Christian Steunou, Claude Zinsou, Wayne Meyers, Michèle Dramaix

**Affiliations:** * Institute of Tropical Medicine, Antwerp, Belgium;; † Centre Sanitaire et Nutritionnel Gbemoten, Zagnanado, Benin;; ‡ Armed Forces Institute of Pathology, Washington, DC, USA;; § Université Libre de Bruxelles, Brussels, Belgium

**Keywords:** Mycobacterium ulcerans, Buruli ulcer, mycobacteriosis, Benin, research

## Abstract

We identified risk factors for Buruli ulcer (BU) in Benin in an unmatched case-control study at the Centre Sanitaire et Nutritionnel Gbemoten in southern Benin. A total of 2,399 persons admitted from 1997 through 2003 and 1,444 unmatched patients with other conditions in 2002 were recruited. Adjusted odds ratios were determined for age, sex, place of residence, *Mycobacterium bovis* BCG vaccination at birth, type of water for domestic use, and occupation. Children <15 years of age and adults >49 years of age had a higher risk for BU. Use of unprotected water from swamps was associated with increased risk for BU; this association was strongest in adults >49 years of age. Sex was not a risk factor for BU. Our data showed that BU was mainly associated with age, place of residence, and water sources in all age groups. Risk for BU was higher in BCG-vaccinated patients >5 years of age.

## Abstract 2

Disease was associated with age, place of residence, and water sources in all age groups.

The World Health Organization (WHO) defines Buruli ulcer (BU) as "an infectious disease involving the skin, caused by *Mycobacterium ulcerans*, characterized by a painless nodule, papule, plaque or edema, evolving into a painless ulcer with undermined edges, often leading to invalidating sequelae. Sometimes bones are destroyed." (*1*). After tuberculosis and leprosy, BU is the third most common mycobacterial disease (*1*). Incidences have increased recently, especially in West Africa (*2**–**6*). In 1997, WHO recognized BU as an emerging public health problem.

Classically, BU is a disease of rural areas and associated with wetlands. Endemic foci exist in tropical Africa, the Americas, Australia, and Asia (*1**,**7**,**8*). Epidemiologic observations concentrate on descriptive information or identification of new foci; however, few case-control studies have been published (*2**,**3**,**9*). Our objective was to assess sociodemographic variables and selected environmental factors associated with BU in Benin.

## Patients and Methods

### Setting

Benin is a West African country with 6,752,569 inhabitants: 51% are female (Third General Census, 2002. unpub. data). The population is young: 48% are <15 years old. Benin was formerly divided into 6 regions (Atlantique, Mono, Borgou, Zou, Oueme, and Atacora); however, each region is now divided into 2 regions (Atlantique/Littoral, Mono/Couffo, Borgou/Alibori, Zou/Colline, Oueme/Plateau, and Atacora/Donga).

The Centre Sanitaire et Nutritionnel Gbemoten (CSNG) in Zou, Benin, is the regional referral center for BU treatment. From 1989 through 2003, CSNG treated >4,000 BU patients. This study included the period 1997–2003. The population of Zou in 2001 was 1,112,943.

### Sample Size

Endemic foci of BU are associated with stagnant bodies of water (ponds, backwaters, and swamps). Data from the Benin Demographic and Health Survey (DHS) 2001 show that 34.5% of the Zou community drink unprotected water (rain water, unprotected well, surface water) (*10*). The sample size calculated with EpiInfo (Centers for Disease Control and Prevention, Atlanta, GA, USA) was 147 cases and 147 hospital controls (control-case ratio 1, odds ratio [OR] > 2, proportion of exposition to unprotected water in controls 34.5%, power 80% [1 – β], and 95% confidence level α = 0.05).

Since we had a large amount of data at our disposal, we analyzed the entire group of case-patients and all controls recruited during 6 months in 2002. The duration of enrollment of controls was limited to 6 months because of problems of availability of the healthcare staff.

### Case-Patient Recruitment

All patients with suspected BU were examined by a physician (C.Z.) and the nurse (J.A.) in charge of the CNSG. Historic and clinical data were recorded on standard forms. One or more of the following features suggested by WHO were used to diagnose BU (*1*): typical nodular, indurated plaque or edematous lesion; >1 painless chronic ulcers with undermined edges or a depressed scar; swelling over a painful joint, suggesting bone involvement; slowly developing lesion (weeks or months); no fever or regional lymphadenopathy; and residence or travel in a zone endemic for BU.

CSNG admitted 2,468 suspected BU patients from 1997 through 2003. Sixty-nine patients were excluded: 56 with recurrent cases and 13 with another definitive diagnosis; 2,399 patients with BU were enrolled in the study.

### Hospital Control Recruitment

In 2002, controls were recruited among patients seen at CSNG for conditions other than BU: 21% had malaria, 6.0% had bronchitis, 5.6% had rheumatism, and 5.5% had parasitosis. The frequency of all other conditions was <5%.

CSNG had 2 types of clinics: 1 for persons <5 years old and 1 for all others. Both clinics interviewed and examined people 3 times a week. For those >5 years old (group 1), the head nurse interviewed all patients with a disease other than BU. For those <5 years old (group 2), the parents or a guardian of 5 subjects were interviewed from each clinic. This method generated an unmatched control group of 1,444 persons.

### Ethics

The Ethical Committee of Benin approved this study. All persons or their mothers or guardians received information about the disease and its treatment. All BU cases and controls received treatment. Verbal consent was obtained from participants or their parents or guardians.

### Data Collection

Data were available from case files; information on the same variables was collected from controls. Data were then analyzed to identify potential risk factors for BU. All data on age, sex, region of residence, occupational activity, *Mycobacterium bovis* BCG scar status, and type of domestic water used were recorded during the clinical examination.

Water sources were categorized into 4 groups: pumped water, river, mixed sources (pumped and river), and swamps. The 4 groups were classified into 2 groups: protected water (pumped) and unprotected water (all other sources). Residences were categorized as Zou, Oueme, and other.

A total of 525 children were in group 1: 339 BU cases and 186 controls. Complete data, except water sources, were available for 339 persons (case-patients and controls). These persons were analyzed by logistic regression. When water source was entered into the model, this number decreased to 246. Age was divided into 3 groups: <1 year, 1–2 years, and 3–4 years. Occupation was not introduced into the model for children <5 years of age.

Group 2 had 3,252 persons: 2,032 BU patients and 1,220 controls. Because data were missing for 930 persons (775 BU patients and 155 controls), 2,322 persons were analyzed by logistic regression. This number was reduced to 1,775 and 1,332 persons, respectively, when water sources and water sources or activities were introduced into the model. Age was divided into 4 groups based on the ORs shown in Table 1: 5–14 years, 15–29 years, 30–49 years, and >50 years. Occupation was grouped as follows: attending or not attending school (for school-age persons), farmer, housewife, and other (e.g., salesperson, tailor, hairdresser, or teacher).

**Table 1:** Distribution of cases of Buruli ulcer and controls by age, Centre Sanitaire et Nutritionnel Gbemoten, Zou, Benin*

Age group, y	Cases (%)	Controls (%)	Total	Crude OR (95% CI)	p value
5–9	432 (21.3)	38 (3.1)	470	6.69 (2.86–16.62)	<0.001
10–14	405 (19.9)	59 (4.8)	464	4.4 (1.77–9.24)	0.002
15–19	223 (11.0)	117 (9.6)	340	1.12 (0.5–2.53)	NS
20–24	163 (8.0)	140 (11.5)	303	0.68 (0.3–1.54)	NS
25–29	149 (7.3)	157 (12.9)	306	0.56 (0.25–1.26)	NS
30–34	84 (4.1)	140 (11.5)	224	0.35 (0.15–0.81)	0.010
35–39	72 (3.5)	136 (11.1)	208	0.31 (0.14–0.72)	0.0046
40–44	44 (2.2)	98 (8.0)	142	0.26 (0.11–0.62)	0.002
45–49	53 (2.6)	81 (6.6)	134	0.38 (0.16–0.9)	0.025
50–54	68 (3.3)	60 (4.9)	128	0.67 (0.28–1.57)	NS
55–59	66 (3.2)	45 (3.7)	111	0.86 (0.36–2.06)	NS
60–64	105 (5.2)	52 (4.3)	157	1.19 (0.51–2.78)	NS
65–69	65 (3.2)	38 (3.1)	103	1.01 (0.42–2.42)	NS
70–74	50 (2.5)	32 (2.6)	82	0.92 (0.37–2.26)	NS
75–79	36 (1.8)	17 (1.4)	53	1.25 (0.47–3.29)	NS
>80	17 (0.8)	10 (0.8)	27	1	
Subtotal	2,032	1,220	3,252		
Missing	367	224	591		
Total	2,399	1,444	3,843		

*OR, odds ratio; CI, confidence interval; NS, not significant.

### Data Analysis

Data were analyzed with SPSS version 9.05 (SPSS Inc., Chicago, IL, USA) and EpiInfo version 6.02. Because of the selection method of controls in the clinic for children, control children <5 years old were underrepresented, and analysis was performed separately for these children. Crude ORs and 95% confidence intervals (CIs) were calculated to evaluate the association between BU and various factors. Contingency tables were analyzed with the Pearson χ^2^ test.

A backward elimination procedure, based on likelihood ratio, was used to select variables to include in logistic regression models. Because age and occupation were correlated (Spearman ρ = 0.626), we introduced either age or occupation into the logistic models. Some interactions were tested in the logistic model including age with the likelihood ratio test. Interaction between age and water source appeared significant; thus, separate logistic models were established for each age category. Because sex and occupation were virtually redundant (e.g., female and housewife), models including occupation were established separately for each sex. Adjusted ORs and 95% CIs were derived from the final logistic models. For these models, goodness of fit was assessed with the Hosmer and Lemeshow test.

## Results

### Group 1

Table 2 shows results of univariate analysis for group 1 (crude ORs and 95% CIs). Age, place of residence, and water sources were associated with BU. Sex was not associated with BU. The risk for BU was ≈3× higher in children without a BCG scar. BU was associated with having a BCG scar (p = 0.047). Risk for BU was particularly high when the water source was a swamp compared with pumped water (OR 47.59, 95% CI 13.76–164.61).

**Table 2:** Univariate analysis of risk factors for Buruli ulcer in children <5 years of age, Centre Sanitaire et Nutritionnel Gbemoten, Zou, Benin*

Variable		Cases (%) (n = 339)	Controls (%) (n = 186)	Crude OR	95% CI	p value
Age, y						<0.001
	<1	31 (9.1)	44 (23.7)	1		
	1–2	154 (45.4)	103 (55.4)	2.12	1.26–3.58	
	3–4	154 (45.4)	39 (21.0)	5.60	3.14–10.00	
Sex						NS
	Female	169 (50.6)	85 (46.4)	1		
	Male	165 (49.4)	98 (53.6)	0.85	0.59–1.22	
*Mycobacterium bovis* BCG scar						0.047
	Present	158 (92.4)	170 (97.1)	1		
	Absent	13 (7.6)	5 (2.9)	2.80	0.98–8.03	
Region						0.001
	Zou	238 (70.2)	152 (81.7)	1		
	Oueme	60 (17.7)	29 (15.6)	1.32	0.81–2.15	
	Other	41 (12.1)	5 (2.7)	5.24	2.02–13.55	
Water sources						<0.001
	Protected (pump)	29 (30.2)	92 (54.8)	1		
	Unprotected (combined types)	67 (69.8)	76 (45.2)	2.80	1.59–4.93	
	River	7 (7.3)	44 (26.2)	0.50	0.21–1.24	
	Unprotected (mixed)	15 (15.6)	29 (17.3)	1.64	0.77–3.47	
	Swamp	45 (46.9)	3 (1.8)	47.59	13.76–164.61	

*OR, odds ratio; CI, confidence interval; NS, not significant.

Table 3 shows results of multivariate analysis for group 1. In this model, age, place of residence, and water sources were associated with BU. The risk for BU was high in children 3–4 years old (OR 6.74, 95% CI 2.67–17.03), those living in Oueme (OR 2.78, 95% CI 1.35–5.70) or other places (OR 9.74, 95% CI 2.27–41.76), and those using unprotected water (OR 2.27, 95% CI 1.22–4.22). Sex and a BCG scar were not associated with BU.

**Table 3:** Multivariate analysis of risk factors for Buruli ulcer in 246 children (87 case-patients and 159 controls) <5 years of age, Centre Sanitaire et Nutritionnel Gbemoten, Zou, Benin*

Variable		Adjusted OR†	95% CI	p value
Age, y				<0.001
	<1	1		
	1–2	1.58	0.62–3.97	
	3–4	6.74	2.67–17.03	
Region				<0.001
	Zou	1		
	Oueme	2.78	1.35–5.70	
	Other	9.74	2.27–41.76	
Water sources				0.010
	Protected	1		
	Unprotected	2.27	1.22–4.22	

*OR, odds ratio; CI, confidence interval. †Adjusted for effects of all variables included in the model. Nonsignificant variables were sex and *Mycobacterium bovis* BCG scar.

### Group 2

Table 1 shows distribution of case-patients and controls by age group. The age groups used (5–14, 15–29, 30–49, and >50 years) were based on the crude ORs found. The 30- to 49-year age group had the lowest risk for BU.

Table 4 shows results of univariate analyses for persons >5 years old. All variables were associated with BU infection. Risk for BU was highest in persons using mixed sources of water (OR 16.72, 95% CI 10.63–26.31). The risk for BU in those using river water was similar to that of those using swamp water. When water sources were classified as protected and unprotected water, the OR was 5.15 (95% CI 4.22–6.28) for those using unprotected water.

**Table 4:** Univariate analysis of risk factors for Buruli ulcer in persons >5 years of age, Centre Sanitaire et Nutritionnel Gbemoten, Zou, Benin*

Variable		Cases (%) (n = 2,032)	Controls (%) (n = 1,220)	Crude OR	95% CI	p value
Age, y						<0.001
	5–14	837 (41.2)	97 (8.0)	15.52	11.96–20.13	
	15–29	535 (26.3)	414 (33.9)	2.32	1.90–2.84	
	30–49	253 (12.5)	455 (37.3)	1		
	>50	407 (20.0)	254 (20.8)	2.88	2.31–3.59	
Sex						<0.001
	Female	986 (49.2)	675 (57.0)	1		
	Male	1,017 (50.8)	510 (43.0)	1.36	1.18–1.57	
*Mycobacterium bovis* BCG scar						<0.001
	Present	969 (75.6)	610 (55.7)	1		
	Absent	313 (24.4)	486 (44.3)	0.41	0.34–0.48	
Region						<0.001
	Zou	1,110 (54.6)	883 (72.4)	1		
	Oueme	575 (28.3)	298 (24.4)	1.53	1.30–1.81	
	Other	347 (17.1)	39 (3.2)	7.08	5.02–9.97	
Water sources						<0.001
	Protected (pump)	296 (36.2)	867 (74.5)	1		
	Unprotected (combined types)	522 (63.8)	297 (25.5)	5.15	4.22–6.28	
	River	75 (9.2)	47 (4.1)	4.67	3.17–6.89	
	Unprotected (mixed)	137 (16.7)	24 (2.1)	16.72	10.63–26.31	
	Swamp	310 (37.9)	226 (19.4)	4.02	3.24–4.99	

*OR, odds ratio; CI, confidence interval.

In the 5–14-year age group, no difference concerning schooling was observed between case-patients and controls. In those >14 years old, more controls were housewives than were case-patients (28.5% vs. 2.6%), and more case-patients were farmers than were controls (49.4% vs. 29.0%).

Univariate analyses by sex and occupation showed that in males, being school age and being a farmer produced the higher risk for BU (data not shown). Young boys (independent of school attendance) had the greatest risk. In females, school-age girls and farmers had similar risks for BU, but housewives had the lowest risk (data not shown).

Table 5 shows results of logistic regression analyses of risk factors for BU in persons >5 years old. Occupation was excluded because it correlated with age. The model included age, BCG scar, place of residence, and water sources but excluded sex (not significant). When adjusted for other variables, children 5–14 years old had a higher risk for BU (OR 11.64, 95% CI 8.01–16.91). The OR (95% CI 3.04–6.27) for BU was 4.36 in those >50 years old. Risk for BU was associated with exposure to unprotected water sources (OR 4.62, 95% CI 3.61–5.90). Absence of a BCG scar decreased the risk for BU (OR 0.40, 95% CI 0.31–0.52). Living a greater distance from CSNG was strongly associated with an increased risk for BU (OR 13.02, 95% CI 8.03–21.11).

**Table 5:** Logistic regression analysis of risk factors for Buruli ulcer in 1,775 persons (754 case-patients and 1,021 controls) >5 years of age, Centre Sanitaire et Nutritionnel Gbemoten, Zou, Benin*

Variable		Adjusted OR†	95% CI	p value
Age, y				<0.001
	5–14	11.64	8.01–16.91	
	15–29	2.21	1.60–3.05	
	30–49	1		
	>50	4.36	3.04–6.27	
*Mycobacterium bovis* BCG scar				<0.001
	Present	1		
	Absent	0.40	0.31–0.52	
Region				<0.001
	Zou	1		
	Oueme	4.02	3.09–5.24	
	Other	13.02	8.03–21.11	
Water sources				<0.001
	Protected	1		
	Unprotected	4.62	3.61–5.90	

*OR, odds ratio; CI, confidence interval. †Adjusted for effects of all variables included in the model. The nonsignificant variable was sex.

Logistic regression showed a strong interaction between age and water source. Thus, a logistic model was produced for each age group. Adjusted ORs and 95% CIs derived from models in the different age groups showed that absence of a BCG scar decreased the risk for BU in all age groups (data not shown). Risk for BU associated with residence of patients was reduced in those >50 years old. BU patients were 1.94× more likely than controls to use unprotected water (OR 1.94, 95% CI 1.20–3.12), and ORs were at least twice those in other age groups (data not shown).

Final results of multivariate analyses by sex and occupation in patients >5 years old showed that the risk for BU was similar in both sexes for the following factors: BCG scar, place of residence, and water sources (data not shown). Risk for BU in boys was strongly associated with school age (OR 4.63, 95% CI 2.63–8.15). This risk was lower in girls (OR 2.75, 95% CI 1.59–4.76). Risk in female farmers was strongly increased (OR 3.51, 95% CI 1.97–6.26). Housewives showed a strong decreased risk for BU (OR 0.06, 95% CI 0.03–0.13).

## Discussion

This is the first large case-control study to describe sociodemographic and environmental factors associated with BU. Our data show that BU was associated with age, place of residence, and water sources for all persons, and that BU was associated with a BCG scar in persons >5 years old.

In accordance with previous studies (*1**–**3**,**11*), the risk for BU was higher in children <15 years old. Children 3–4 years old had an increased risk for BU if they used unprotected water and lived in areas endemic for BU. That clothing provides protection against BU has already been reported in Côte d'Ivoire (*3*). In Africa, infants <1 year of age are often protected by clothes, bonnets, and booties. At ≈1–2 years of age, children begin to walk and play in dirt near their home, and those 3–4 years of age are more independent and roam freely in the environment, usually scantily clothed, which increases exposure to a contaminated environment. Use of unprotected water increases BU risk (OR >40), especially in children <5 years old. These associations have been previously reported (*9**,**12**,**13*). Molecular studies showed *M*. *ulcerans* in water, mud, fish, aquatic insects, and snails from swamps in regions endemic for BU (*14**–**18*). Material from swamps, ponds, or river regions may contaminate skin surfaces with *M*. *ulcerans*, which can result in introduction of the causative agent into skin when it is broken by trauma or insect bites (*15**,**19*).

Children 5–14 years old and persons >49 years old were at higher risk for BU. Their attire and contact with environmental sources of *M*. *ulcerans* may be a relevant factor. Elderly persons through repeated episodes of exposure to *M*. *ulcerans* may acquire latent infections that are reactivated by age-related immunosuppression. Results from the logistic model in the >50-year age group support this concept. In this age group, risk for BU showed a lower correlation with unprotected water than in other age groups.

Although adults 15–49 years old are frequently exposed to wetlands, their risk for BU was lower, which suggests acquired resistance to the disease. We speculate that this resistance may be related to acquired specific immunity or to cross-immunity from other mycobacterioses (*20*).

We previously reported that men >59 years old in Zou had a higher risk for BU than women, but men and women in the <59-year-old group were equally at risk (*11*). Our present study did not confirm sex differences in either those >50 or <50 years of age. These 2 studies differ in design: the earlier study (*11*) of the general population of Zou showed that 43% were boys and men. In this study, the percentage of male control subjects was slightly higher (47%) and may suggest that no differences in risk exist. The percentages of school-age and nonschool-age male BU patients increased relative to controls, while the percentages of female farmers and school-age and nonschool-age girls were nearly equal. Although we did not evaluate specific work or duties, girls often carry water and perform other tasks, but young boys usually play and frequent contaminated environments. Reduced risk for BU in housewives may be related to their reduced contact with contaminated environments. Differences between age and occupation may reflect differences in age-specific frequency and intensity of exposure to *M*. *ulcerans*.

From 1997 to 2001, CSNG received patients mainly from the Zou Region (*6*). Few patients come to CSNG from remote areas for treatment of diseases other than BU. The total number of BU patients is likely underestimated because of complex socioeconomic factors involved in care seeking and treatment of chronic diseases such as BU (*21*).

Studies that evaluated the presence or absence of BCG scars to determine vaccination status reported that scars develop in most vaccinated persons (*22**,**23*). No association was found between BU and BCG status in children <5 years old after adjustment for age. In persons >5 years old, a BCG scar resulted in a risk factor of 2.5 for BU compared with those without a BCG scar (Table 5). Our results differ from those of 2 prospective studies that reported that BCG vaccination partially protects against BU (*24**,**25*). However, this protection seemingly decreased after 6 months (*24*).

In children in Benin >5 years old, a BCG scar may represent a risk factor for BU. Vaccination may rarely introduce *M*. *ulcerans* intradermally from contaminated skin (*26*). At CSNG, BU developed in several patients at the site of BCG vaccination (J. Aguiar, C. Steunou, C. Zinsou, unpub. data).

The efficacy of BCG in preventing dissemination of tuberculosis in children is well known (*27*). BCG vaccination at birth also provides protection against the development of *M*. *ulcerans* osteomyelitis (*28**,**29*). The disparity in the results of our case-control study may reflect known variations of efficacy of BCG (*30*). Factors related to host and the causative agent may explain varying protection of BCG against BU (*31*). An alternative explanation is variation of BCG coverage for controls (general population or hospital controls) compared with BU patients (*32*). From 1993 to 1999, BCG vaccination coverage in Zou was stable (≈90%–100%). A matched case-control study in Benin of neighborhood controls and BU cases to assess the efficacy of BCG vaccination at birth is in progress. This ongoing study will evaluate the validity of our findings (*33*).

Cases and controls are likely to have similar equal exposures to environmental transmission factors for BU. Results of our study are consistent when comparing data from other regions in Benin or only from Zou.

This case-control study confirms findings of previous studies, which indicated that children <15 years of age are at highest risk for acquiring BU (*1**,**6*), and that in areas endemic for BU, exposure to unprotected water is a risk factor for the disease (*1**,**14*). Our study demonstrates that the association between BU and exposure to unprotected water is not as strong in patients >50 years of age than in other age groups. This suggests that BU in older people may be related to reactivation of latent infections by *M*. *ulcerans*. This study also demonstrates differences between sexes, which may be associated with age-influenced domestic, agricultural, and recreational activities. BCG vaccination is a risk factor for BU in persons >5 years old. Because of the diversity of conditions encountered among controls, we do not believe that these conditions have influenced our results.

Programs for provision of protected water for drinking and domestic use would have the greatest effect on control of BU in Benin. This effect could be accomplished by drilling wells and supplying pumps. Appropriate educational programs that promote behavioral changes should also reduce the frequency of BU.
